# Higher oxidative balance score decreases risk of stroke in US adults: evidence from a cross-sectional study

**DOI:** 10.3389/fcvm.2023.1264923

**Published:** 2023-11-14

**Authors:** Fangfang Zhan, Gaoteng Lin, Kefei Duan, Bixia Huang, Longfei Chen, Jun Ni

**Affiliations:** ^1^ Department of Rehabilitation Medicine, The First Affiliated Hospital of Fujian Medical University, Fuzhou, China; ^2^ Department of Rehabilitation Medicine, National Regional Medical Center, Binhai Campus of the First Affiliated Hospital, Fujian Medical University, Fuzhou, China; ^3^ Department of Urology, The 900th Hospital of Joint Logistic Support Force, Fuzhou, China; ^4^ Department of Geriatric Medicine, Tianjin Medical University General Hospital, Tianjin, China; ^5^ Department of Neurology, The Affiliated Hospital of Putian University, Putian, China; ^6^ Department of Neurology, National Regional Medical Center, Binhai Campus of the First Affiliated Hospital, Fujian Medical University, Fuzhou, China; ^7^ Department of Neurology, The First Affiliated Hospital of Fujian Medical University, Fuzhou, China

**Keywords:** oxidative balance score, stroke, antioxidants and prooxidants, diet, lifestyle

## Abstract

**Background:**

The oxidative balance score (OBS) can be used to represent the overall burden of oxidative stress in an individual. This study aimed to explore the association between the risk of stroke and OBS.

**Methods and materials:**

The National Health and Nutrition Examination Survey (NHANES) from 1999 to 2018 was used to extract a series of variables for participants who took the stroke questionnaire. The construction of OBS relied on diet and lifestyle components, which included 16 nutrients and 4 lifestyle factors. Weighted multivariable-adjusted logistic regression was performed to investigate the association between stroke risk and OBS. A stratified analysis was also conducted. The dose-response relationship between stroke risk and OBS was elucidated by performing a restricted cubic spline function.

**Results:**

A total of 20,680 participants were included for analysis, 768 of whom suffered from stroke. Based on weighted multivariable logistic regression analysis, we discovered that the stroke prevalence decreased by 2% for each OBS unit added [OR: 0.98 (0.97–1.00), *P* < 0.01]. For the OBS subgroup, we also discovered that higher OBS was related to a reduction in the risk of stroke [Q4 vs. Q1: OR:0.65 (0.46–0.90), *P* < 0.01]. The prevalence of stroke declined by 3% with every OBS unit added to the diet component [OR: 0.97 (0.96–0.99), *P* < 0.01]. For the dietary OBS subgroup, higher OBS in diet components was associated with a decrease in the prevalence of stroke [Q4 vs. Q1: OR: 0.65, (0.47–0.91), *P* < 0.05]. Further stratified analysis showed that every OBS unit raised was associated with a decline in stroke prevalence, which was statistically significant in participants in subgroups of ≥60 years, female, no-diabetes mellitus and no-hypertension. OBS and stroke prevalence were correlated in a linear manner.

**Conclusion:**

The study found that a higher OBS was associated with a decrease in stroke prevalence, which could be a significant indicator for evaluating stroke risk.

## Introduction

The number of stroke events worldwide was estimated by the Global Stroke Epidemiology to be 16.9 million in 2010. The number of new stroke cases in 2016 reached 13.7 million, and the global stroke prevalence was 80.1 million. Stroke caused the death of 5.5 million people worldwide in the same year (1, 2). Stroke is characterized by high incidence, high disability rate, high mortality rate, and high recurrence rate. The patients' families endure significant financial losses and physical and mental pain, which has resulted in significant strains on the individual and social levels (3, 4). Although stroke incidence has been on the rise in the aging population, most strokes can be prevented by controlling risk factors and early intervention. Therefore, finding risk factors has become the primary concern of patients and physicians.

The oxidative balance score (OBS) is a way to measure exposure to antioxidants and prooxidants in diet and lifestyle, which represent the overall burden of oxidative stress (5). Numerous studies have shown that OBS is correlated with various chronic diseases, including type 2 diabetes (6), osteoarthritis (7), chronic kidney disease (8), cardiovascular disease (9), and cancer (10, 11). At the same time, OBS can be used as a predictor of all-cause mortality, cancer mortality, and non-cancer mortality, which was put forward by the data of a large-scale national prospective cohort study. OBS has the potential to be a valuable tool in evaluating the impact of lifestyle and dietary factors on oxidative stress. The risk of premature all-cause, cancer, and non-cancer deaths may be decreased by a higher OBS (12). However, the relationship between OBS and stroke is still unclear, and there is no study to evaluate the relationship between comprehensive exposure to lifestyle and dietary factors and stroke. Therefore, the purpose of this study is to evaluate the relationship between OBS and stroke risk through a cross-sectional study.

## Methods and materials

### Sample

The National Health and Nutrition Examination Survey (NHANES), a cross-sectional population survey, focuses on the health and nutrition status of adults and children in the United States, which is administrated by the Centers for Disease Control and Prevention (CDC). The protocol was approved by the National Center for Health Statistics Ethics Review Board and written informed consent was obtained from all participants. In this study, the data materials for participants involved in the stroke questionnaire from 1999 to 2018 were collected from NHANES (https://www.cdc.gov/nchs/nhanes/index.htm). The question about stroke was: “Has a doctor or other health professional ever told you that you had a stroke?” (outcome). The stroke participants were defined when they answered “Yes” to the question. The questionnaire was completed by 52,740 participants, with 2,265 of them suffering from stroke.

### Oxidative balance score (exposure)

The construction and calculation of OBS have been elaborated upon in previous studies (13). The construction of OBS was based on diet and lifestyle components, which included 16 nutrients and 4 lifestyle factors. The 20 components were further classified into pro-oxidants (total fat, iron, alcohol intake, BMI, and cotinine) and antioxidants (dietary fiber, β-carotene, vitamin B_2_, niacin, vitamin B_6_, total folate, vitamin B_12_, vitamin C, vitamin E, calcium, magnesium, zinc, copper, selenium, and physical activity). Each component of diet and lifestyle was given a score based on its property (antioxidant or prooxidant) and gender (men or women) according to the OBS components' assignment scheme. The total score for OBS was the sum of scores for each part. A higher OBS represented a predominance of antioxidant exposure.

The assignment scheme for the OBS components was created based on previous studies (13). Especially, according to alcohol consumption level, participants were divided into non-drinkers, non-heavy drinkers (0 to 15 g/d for women and 0 to 30 g/d for men), and heavy drinkers (≥15 g/d for women and ≥30 g/d for men), assigning 2, 1, and 0 points, respectively (13). Other components were grouped through tertiles based on gender. The antioxidants groups scored 0 to 2 points from tertile 1 to tertile 3, respectively, whereas in prooxidants groups, tertile 3 was designated as 0 point and tertile 1 as 2 points.

### Covariates assessments

The final outcome was adjusted by incorporating a variety of covariates. General information obtained from questionnaires included age (<60 and ≥60 years), sex (male or female), race (non-Hispanic white, non-Hispanic Black, Hispanic-Mexican, and others), and smoking status (never, ever, and now). The smoking status was divided based on whether participants have smoked at least 100 cigarettes in their lifetime and whether they smoke now. Biochemical indicators contained serum cholesterol (mmol/L), LDL-cholesterol (mmol/L), triglycerides (mmol/L), bilirubin (umol/L), creatinine (umol/L), globulin (g/L), iron (umol/L), glucose (mmol/L), and body mass index (BMI) (kg/m^2^). BMI was calculated by dividing weight by height squared and dividing it into normal weight (<25 kg/m^2^), overweight (25–30 kg/m^2^), and obesity (>30 kg/m^2^). Co-morbidities consisted of diabetes mellitus (yes/no), hypertension (yes/no), and chronic kidney disease (CKD) (yes/no). The definition of diabetes mellitus was either by performing anti-hyperglycemic therapy or by a doctor or health professional (14). Hypertension was identified as taking antihypertensive medication and an average systolic BP ≥ 140 mmHg or diastolic BP ≥ 90 mmHg at baseline (14). CKD was recognized by the urine albumin-to-creatinine ratio (ACR) ≥ 30 mg/g and/or eGFR < 60 ml/min/1.73 m^2^ (15). The Chronic Kidney Disease Epidemiology Collaboration algorithm was performed to calculate the eGFR scores (16).

### Statistical analysis

Continuous variables were shown as median ± interquartile range (IQR). Categorical variables were shown as frequency (%). Survey analysis in 1999–2002 cycles was weighted by “Full Sample 4 Year MEC Exam Weight (wtmec4yr)”, while in 2003–2018, it was weighted by “Full Sample 2 Year MEC Exam Weight (wtmec2yr)”. OBS was considered to be a continuous variable. OBS group (quartile conversion) was regarded as a categorical variable. The relationship between OBS and the prevalence of stroke was investigated using univariable and weighted multivariable logistic regression analysis adjusted for different covariates. The crude model was based on univariable logistic regression. Model 1 was built on multivariable weighted logistic regression adjusted for age, gender, and race. Model 2 was adjusted for age, gender, race, serum cholesterol, LDL-cholesterol, triglycerides, bilirubin, creatinine, globulin, iron, glucose, BMI, diabetes mellitus, hypertension, and CKD. Then, restricted cubic spline (RCS) with three knots located at the 33.33th, 66.66th, and 99.99th percentiles was performed to investigate the dose-response association between OBS and stroke prevalence. A stratified analysis was also conducted to reflect the difference in stroke prevalence among different groups. All analyses were performed in R software (version: 4.2.2) by using the “nhanesR” package. The “survey” package was employed to carry out weighted logistic regression analysis. *P* < 0.05 was considered to be statistically significant.

## Results

### The baseline characteristics of NHANES participants from 1999 to 2018

The baseline characteristics of NHANES participants with/without stroke from 1999 to 2018 are displayed in Table 1. After combining with covariates, a total of 20,680 participants with complete information were included in the analysis, of which 768 participants suffered from stroke. There were 200 stroke patients who were under 60 years old and 568 stroke patients who were over 60 years old. In the gender grouping, 380 patients were men and 388 patients were women. Quartile conversion was performed for continuous OBS, followed by subgroup determination (Q1, Q2, Q3, and Q4). The range score for each OBS subgroup was 0–11, 11–18, 18–25, and 25–37, respectively. The samples for each OBS subgroup (Q1, Q2, Q3, and Q4) were 5,485, 4,980, 5,764, and 4,451, respectively. The assignment scheme of the OBS components is listed in Table 2.

**Table 1:** Baseline characteristics of NHANES participants (1999–2018) by stroke for OBS.

Characteristics	*N* (%)	No history of stroke	History of stroke
All participants	20,680 (100.00)	19,912 (96.3)	768 (3.7)
Categorical variables			
Age (years)			
<60	13,517 (65.4)	13,317 (98.5)	200 (1.5)
≥60	7,163 (34.6)	6,595 (92.1)	568 (7.9)
Gender			
Male	10,228 (49.5)	9,848 (96.3)	380 (3.7)
Female	10,452 (50.5)	10,064 (96.3	388 (3.7)
Race			
Non-Hispanic White	9,263 (44.8	8,872 (95.8)	391 (4.2)
Non-Hispanic Black	4,106 (19.8)	3,907 (95.2)	199 (4.8)
Mexican American	3,611 (17.5)	3,522 (97.5	89 (2.5)
Other race	3,700 (17.9)	3,611 (97.6)	89 (2.4)
BMI (kg/m^2^)			
<25	6,306 (30.5)	6,116 (97.0)	190 (3.0)
25–30	6,988 (33.8)	6,745 (96.5)	243 (3.5)
≥30	7,386 (35.7)	7,051 (95.5)	335 (4.5)
Smoke			
Never	11,154 (53.9)	10,859 (97.4)	295 (2.6)
Former	5,219 (25.3)	4,930 (94.5	289 (5.5)
Now	4,307 (20.8)	4,123 (95.7)	184 (4.3)
Hypertension			
Yes	8,774 (42.4)	8,168 (93.1)	606 (6.9)
No	11,906 (57.6)	11,744 (98.6)	162 (1.4)
Diabetes			
DM	2,594 (12.5)	2,360 (91.0)	234 (9.0)
No	18,086 (87.5)	17,552 (97.0)	534 (3.0)
CKD			
Yes	3,646 (17.6)	3,292 (90.3)	354 (9.7)
No	17,034 (82.4)	16,620 (97.6)	414 (2.4)
OBS group			
Q1	5,485 (26.5)	5,204 (94.9)	281 (5.1)
Q2	4,980 (24.1)	4,766 (95.7)	214 (4.3)
Q3	5,764 (27.9)	5,581 (96.8)	183 (3.2)
Q4	4,451 (21.5)	4,361 (98.0)	90 (2.0)
Continuous variables			
Serum cholesterol	4.94 ± 1.37	4.94 ± 1.35	4.71 ± 1.51
Serum LDL-cholesterol	113 ± 47	113 ± 46	104 ± 53
Serum triglycerides	1.17 ± 0.89	1.16 ± 0.89	1.30 ± 0.89
Serum bilirubin	11.97 ± 5.13	11.97 ± 5.13	10.3 ± 5.13
Serum creatinine	73.37 ± 26.52	72.49 ± 26.52	87.52 ± 36.24
Serum globulin	29 ± 6	29 ± 6	30 ± 6
Serum iron	15.2 ± 8	15.2 ± 8.1	13.8 ± 7
Serum glucose	5.22 ± 0.94	5.22 ± 0.89	5.55 ± 1.39

NHANES, the National Health and Nutrition Examination Survey; OBS: oxidative balance score; CKD, chronic kidney disease;.

Categorical variables were showed by count (percentage), while continuous variables were displayed by median ± interquartile range (IQR).

**Table 2:** Oxidative balance score assignment scheme (*N* = 20,680).

OBS components	Property	Male			Female		
		0	1	2	0	1	2
Dietary OBS components							
Dietary fiber (g/d)	A	<12.85	12.85–20.5	≥20.5	<10.85	10.85–16.8	≥16.8
β-Carotene (RE/d)	A	<103.94	103.94–323.61	≥323.61	<102.15	102.15–349.94	≥349.94
Vitamin B2 (mg/d)	A	<1.73	1.73–2.55	≥2.55	<1.35	1.35–1.96	≥1.96
Niacin (mg/d)	A	<21.42	21.42–31.15	≥31.15	<15.58	15.58–22.57	≥22.57
Vitamin B6 (mg/d)	A	<1.64	1.64–2.46	≥2.46	<1.24	1.24–1.83	≥1.83
Total folate (mcg/d)	A	<320.6	320.6–490.23	≥490.23	<253.5	253.5–380.0	≥380.0
Vitamin B12 (mcg/d)	A	<3.46	3.46–6.1	≥6.1	<2.46	2.46–4.38	≥4.38
Vitamin C (mg/d)	A	<41.7	41.7–102.0	≥102.0	<39.5	39.5–91.78	≥91.78
Vitamin E (ATE) (mg/d)	A	<5.54	5.54–8.90	≥8.90	<4.59	4.59–7.51	≥7.51
Calcium (mg/d)	A	<683.5	683.5–1087.5	≥1087.5	<570.0	570.0–881.7	≥881.7
Magnesium (mg/d)	A	<249.0	249.0–355.5	≥355.5	<199	199–280	≥280
Zinc (mg/d)	A	<9.58	9.58–14.21	≥14.21	<7.01	7.01–10.22	≥10.22
Copper (mg/d)	A	<1.03	1.03–1.49	≥1.49	<0.84	0.84–1.20	≥1.20
Selenium (mcg/d)	A	<97.6	97.6–141.3	≥141.3	<72.0	72.0–103.6	≥103.6
Total fat (g/d)	P	≥100.30	66.80–100.30	<66.80	≥75.04	50.18–75.04	<50.18
Iron (mg/d)	P	≥18.47	12.50–18.47	<12.50	≥14.01	9.67–14.01	<9.67
Lifestyle OBS components							
Physical activity (MET-minute/week)	A	<690.73	690.73–3360.0	≥3360.0	<520	520–1920	≥1,920
Alcohol (drinks/d)	P	≥30	0–30	None	≥15	0–15	None
Body mass index (kg/m^2^)	P	≥29.76	25.49–29.76	<25.49	≥31.3	25.3–31.3	<25.3
Cotinine (ng/ml)	P	≥2.38	0.03–2.38	<0.03	≥0.12	0.019–0.12	<0.019

The dietary components did not include nutrients obtained from dietary supplements or medications.

OBS, oxidative balance score; A, antioxidant; P, prooxidant; RE, retinol equivalent; ATE, alpha-tocopherol equivalent; MET, metabolic equivalent.

### Association between OBS and stroke prevalence

The study to investigate the relationship between OBS and stroke prevalence was based on the crude model, model 1, and model 2, constructed by weighted logistic regression analysis. Table 3 displays the results from the three models. Due to model 2 being adjusted for a range of covariates, the results were more robust and were selected as the report. We found that the results maintained relative stability across three models. As shown in Table 3, the results from model 2 showed that the prevalence of stroke decreased by 2% with each OBS unit added [OR: 0.98 (0.97–1.00), *P* < 0.01]. For OBS subgroups, we also discovered higher OBS was related to a reduction of the prevalence of stroke [Q4 vs. Q1: OR: 0.65 (0.46–0.90), *P* < 0.01], compared with that in quartile 1. The construction of OBS, which was based on diet and lifestyle components, resulted in them having their own scores. As shown in Table 3, we found that the prevalence of stroke declined by 3% with every OBS unit added to the diet component [OR: 0.97 (0.96–0.99), *P* < 0.01]. For dietary OBS subgroups, higher OBS in diet component was associated with a decrease in the prevalence of stroke [Q4 vs. Q1: OR: 0.65, (0.47–0.91), *P* < 0.05], compared with that in quartile 1. However, lifestyle OBS did not appear to be linked to stroke prevalence (Table 3).

**Table 3:** Association between the OBS and stroke prevalence based on weighted logistic regression analysis.

		Odds ratio (95% CI)	
Characteristic	Crude model	Model 1	Model 2
OBS	0.97 (0.96–0.98)****	0.97 (0.96–0.98)****	0.98 (0.97–1.00)**
OBS subgroups			
Q1	ref	ref	ref
Q2	0.88 (0.70–1.11	0.81 (0.64–1.03)	0.88 (0.68–1.15)
Q3	0.69 (0.52–0.92)**	0.68 (0.51–0.90)**	0.82 (0.61–1.11)
Q4	0.44 (0.33–0.59)****	0.45 (0.33–0.61)****	0.65 (0.46–0.90)**
OBS (Dietary components)			
	0.95 (0.94–0.96)****	0.96 (0.94–0.97)****	0.97 (0.96–0.99)**
OBS subgroups (Dietary components)			
Q1	ref	ref	ref
Q2	0.85 (0.65–1.11)	0.88 (0.67–1.15)	0.99 (0.75–1.31)
Q3	0.58 (0.43–0.79)***	0.64 (0.46–0.87)**	0.78 (0.56–1.09)
Q4	0.43 (0.32–0.57)****	0.49 (0.36–0.67)****	0.65 (0.47–0.91)*
OBS (Life components)			
	0.84 (0.79–0.89)****	0.83 (0.78–0.88)****	0.94 (0.87–1.02)
OBS subgroups (Life components)			
Q1	ref	ref	ref
Q2	0.67 (0.53–0.85)**	0.65 (0.51–0.84)***	0.83 (0.63–1.10)
Q3	0.61 (0.46–0.80)***	0.58 (0.44–0.78)***	0.79 (0.58–1.09)
Q4	0.43 (0.31–0.61)****	0.41 (0.30–0.58)****	0.72 (0.48–1.08)

OBS, oxidative balance score; CI, confidence interval; Crude Model, based on weighted univariable logistic regression analysis; Model 1, based on weighted multivariable logistic regression analysis adjusted for age, gender, and race; Model 2, based on weighted multivariable logistic regression analysis adjusted for age, gender, race, serum cholesterol, LDL-cholesterol, triglycerides, bilirubin, creatinine, globulin, iron, glucose, BMI, diabetes mellitus, hypertension, and chronic kidney disease.

* <0.05.

** <0.01.

*** <0.001.

**** <0.0001.

### A stratified analysis of the prevalence of OBS and stroke

Age, diabetes mellitus, and hypertension were known to be important risk factors for stroke prevalence. In this section, we aimed to investigate the effect of momentous variables on the association between OBS and stroke prevalence using stratified analysis. As shown in Table 4, the increase in OBS units was associated with a decrease in stroke prevalence among participants in subgroups of ≥60 years [OR: 0.97 (0.96–0.99), *P* < 0.001], women [OR: 0.98 (0.96–0.99), *P* < 0.01], non-Hispanic white [OR: 0.98 (0.97–1.00), *P* < 0.05], non-Hispanic Black [OR: 0.98 (0.96–1.00), *P* < 0.05], BMI (25–30 kg/m^2^) [OR: 0.97 (0.95–0.99), *P* < 0.05], no hypertension [OR:0.97 (0.95–0.99), *P* < 0.01], no DM [OR: 0.99 (0.97–1.00), *P* < 0.05], and CKD [OR: 0.98 (0.96–0.99), *P* < 0.01]. Although the risk of stroke prevalence decreased with per OBS unit raised in the smoking subgroups, hypertension, DM, and no CKD, the statistical difference was not significant. The interaction analysis showed that the differences between subgroups of each variable were not significant (Table 4).

**Table 4:** Stratified analysis for the association between OBS and stroke prevalence in US adults, NHANES 1999–2018.

Variables	Odds ratio (95% CI)	P for interaction
Age (years)		
<60	1.00 (0.98–1.02)	
≥60	0.97 (0.96–0.99)***	0.11
Gender		
Male	1.00 (0.98–1.01)	
Female	0.98 (0.96–0.99)**	0.12
Race		
Non-Hispanic white	0.98 (0.97–1.00)*	
Non-Hispanic black	0.98 (0.96–1.00)*	0.98
Hispanic-Mexican	0.98 (0.96–1.01)	0.77
Other	1.00 (0.97, 1.03)	0.48
BMI (kg/m^2^)		
<25	1.00 (0.97–1.02)	
25–30	0.97 (0.95–0.99)*	0.22
≥30	0.98 (0.97–1.00)	0.27
Smoke		
Never	0.99 (0.97–1.00)	
Former	0.98 (0.96–1.00)	0.71
Now	0.98 (0.96–1.01)	0.97
Hypertension		
Yes	0.99 (0.98–1.00)	
No	0.97 (0.95–0.99)**	0.07
Diabetes mellitus		
Yes	0.98 (0.95–1.00)	
No	0.99 (0.97–1.00)*	0.66
Chronic kidney disease		
Yes	0.98 (0.96–0.99)**	
No	0.99 (0.97–1.00)	0.36

P for interaction was calculated by Model 2, which was based on weighted multivariable logistic regression analysis adjusted for age, gender, race, serum cholesterol, LDL-cholesterol, triglycerides, bilirubin, creatinine, globulin, iron, glucose, BMI, diabetes mellitus, hypertension, and chronic kidney disease.

OBS, oxidative balance score; CI, confidence interval;.

* <0.05.

** <0.01.

*** <0.001.

**** <0.0001.

### Dose-response association between OBS and stroke prevalence

**Figure 1:**
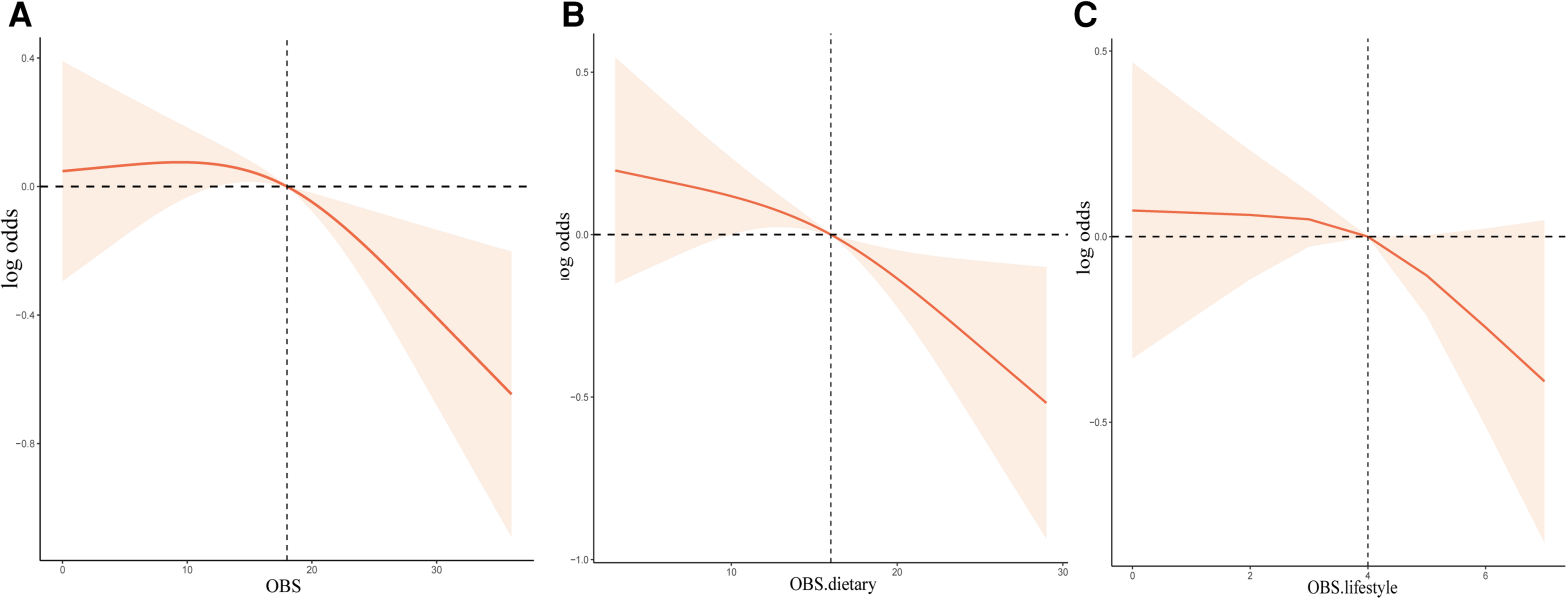
The dose-response association between OBS and the risk of stroke based on RCS analysis. (**A–C**) RCS analysis based on weighted multivariable logistic regression after adjusting covariates to investigate the linear association between OBS, OBS in dietary components, and OBS in lifestyle components and stroke prevalence.

From RCS analysis based on weighted multivariable logistic regression adjusting covariates, we found that there was a linear association between OBS, OBS in dietary components, and OBS in lifestyle components and the risk of stroke prevalence by the spline smoothing plot (P _non−linear_ > 0.05) (Figures 1A–C). The result manifested that higher OBS was related to lower stroke prevalence.

## Discussion

We studied the association between OBS and stroke prevalence and discovered that adding an OBS unit (which reflects an increased antioxidant effect) was associated with a decrease in stroke prevalence. OBS Q4 was associated with a decrease in stroke prevalence among OBS subgroups. In addition, a decrease in stroke risk was found to be associated with OBS in diet components. Further stratified analysis showed that every OBS unit raised associated with a declined risk of stroke prevalence was statistically significant in participants in subgroups of ≥0 years, women, non-Hispanic white, non-Hispanic Black, BMI (25–30 kg/m^2^), no hypertension, no DM, and CKD. The RCS analysis illustrated linear associations between OBS and stroke prevalence. To the best of our knowledge, our research is the first comprehensive retrospective study to investigate the association between OBS and stroke prevalence. It may be considered a good indicator of stroke prevalence.

Many diet ingredients have been shown to be associated with stroke. Dong et al. found that those who eat more dietary fiber were less likely to suffer from stroke (17). A meta-analysis suggested to add the consumption of fiber-rich foods to prevent stroke (18). Dietary β-carotene was found to be related to a reduction in stroke RRs, with a non-linear association (19). Surprisingly, the paper did not conclude that dietary vitamin E was not significantly associated with stroke. Poor B vitamin intake (folate, vitamin B12, and vitamin B6) status can result in high Hcy, which is a risk factor for stroke (20). Vitamin C is an extremely effective antioxidant that is associated with an 11% reduction in stroke prevalence. Keener et al. (21) indicated that niacin can prevent stroke by raising the level of high-density lipoprotein and can be used for the treatment of stroke patients with low serum HDL. Dietary calcium, magnesium, and selenium intake might play an effective role in the prevention of stroke (22–24). However, the relationship between dietary calcium intake and dietary magnesium intake and stroke needs to be investigated further. Ghasemi et al. (25) have discovered that men who have a high iron intake have a statistically significant higher risk of stroke. Owing to the majority of antioxidants in the dietary components, dietary OBS was shown to be inversely linked with stroke prevalence. It was widely accepted that higher fat intake and smoking were risk factors for stroke, while active exercises were inversely correlated with stroke prevalence. The risk of stroke was positively correlated with BMI and lowering BMI can be used as a way to prevent stroke (26), which was consistent with our finding. Smyth et al. (27) concluded that high and moderate alcohol intake was associated with increased odds of stroke, whereas lower intake was not associated with stroke. Therefore, alcohol consumption may be an unfavorable factor for stroke prevalence. What is the way to combine the effects of these factors on stroke? OBS responds to the question. OBS is a reflection of an individual's overall oxidative stress burden (5). Oxidative stress has been identified as a potential therapeutic target for neurological diseases including stroke (28). It is involved in the early stage of stroke, indicating that free radicals play an important role in the development of the disease. The effects of scavenger enzymes and protective anti-oxidants were inhibited by free radicals in ischemic stroke, resulting in cerebral ischemia–reperfusion (I/R) injury. In addition, free radicals also caused mitochondrial dysfunction, apoptotic cascade, and signal transduction pathways which may finally lead to the death of neural cells. The release of proinflammatory cytokines and chemokines, which were involved in the process of the disease, was also caused by oxidative stress in the brain. Thus, preventing the production of free radicals through antioxidant therapy is a feasible strategy for people at high risk of stroke or stroke patients. It is worth noting that the antioxidant effect of the food source is an important source of antioxidant effect *in vivo* (29). A result from Mendelian randomization analysis revealed that genetically proxied circulating *γ*-tocopherol (Vitamin E) was causally associated with total stroke (OR:0.68) (30). Our study demonstrated that OBS in the diet component was associated with a decrease in stroke prevalence. These results further demonstrated the importance of OBS in assessing antioxidant capacity in high-risk stroke patients or those at risk of stroke.

Our study showed that compared to men, every OBS unit raised associated with a declined risk of stroke prevalence was statistically significant in female participants. On the one hand, lifestyle habits (more fruits and vegetables being consumed by women) may contribute to the difference. On the other hand, hormone differences between them may be another important factor. The elderly with ≥60 years observed higher OBS related to lower stroke prevalence, reminding us to adjust dietary structure and lifestyle to reduce risk. Hypertension and DM were the significant risk factors for stroke. Lee et al. (31) indicated that individuals with high OBS are at a lower risk of developing new-onset hypertension. Interestingly, we discovered that per unit added OBS related to the reduction of stroke of participants was statistically significant in no hypertension and no DM groups, while it was not statistically significant in hypertension and DM groups. There was no difference in the two subgroups according to interaction analysis. In other words, preventing stroke in non-hypertensive and non-DM populations requires reasonable diet and lifestyle habits. Chronic kidney disease is a risk factor for stroke and is correlated to poor prognosis (32). However, there was a lack of interventions to prevent and treat stroke in CKD patients. Our findings may offer a feasible strategy.

There were some limitations in the study. Firstly, the cross-sectional study design made it difficult to investigate the causal relationship between oxidative status and stroke. Secondly, stroke encompasses both ischemic and hemorrhagic strokes. Stroke was self-reported through questionnaires or interviews and was not specifically classified in our study. Thirdly, we could not completely adjust or exclude other unknown covariates. Fourthly, owing to the differences in the diet and lifestyle between Western countries and non-Western countries, the results need further verification in non-Western countries.

## Conclusion

The study discovered that the OBS that comprehensively reflects an individual's overall burden of oxidative stress was related to stroke prevalence. Increased OBS was associated with a decrease in stroke prevalence, especially in participants in subgroups of ≥60 years, women, non-Hispanic white, non-Hispanic Black, BMI (25–30 kg/m^2^), no hypertension, no DM, and CKD. OBS and stroke were associated in a linear manner. Therefore, the OBS can be used as an important indicator to assess stroke prevalence.

## Data Availability

Publicly available datasets were analyzed in this study. This data can be found here: Centers for Disease Control and Prevention (CDC), National Center for Healh Statistics (NCHS), National Health and Nutrition Examination Survey (NHANES), https://wwwn.cdc.gov/nchs/nhanes/Default.aspx, NHANES 1999–2018.
